# Recovery From Central Retinal Artery Occlusion Accompanying Paracentral Acute Middle Maculopathy After COVID-19 Vaccination

**DOI:** 10.7759/cureus.51501

**Published:** 2024-01-02

**Authors:** Azusa Yamagishi, Yuta Kitamura, Takayuki Baba

**Affiliations:** 1 Department of Ophthalmology and Visual Science, Chiba University Graduate School of Medicine, Chiba, JPN

**Keywords:** paracentral acute middle maculopathy, covid-19 vaccine, pfizer-biontech covid-19 vaccine, retinal artery occlusion, mrna vaccine, covid-19

## Abstract

Although ocular complications following COVID-19 vaccination have been reported, particularly retinal vascular occlusion and uveitis, their definitive causal relationships remain uncertain. This report presents a case of central retinal artery occlusion (CRAO) with paracentral acute middle maculopathy (PAMM) developed one day after receiving Pfizer COVID-19 vaccine, with a favorable outcome.

The patient experienced sudden vision loss in her left eye, and her vision dropped to hand motion the day after vaccination. The initial examination suggested CRAO, but optical coherence tomography (OCT) revealed PAMM. We administered intravenous d-mannitol and acetazolamide and performed ocular massage. Two days later, her corrected visual acuity improved to 0.4, and further improvement to 1.2 occurred after 16 days.

To the best of our knowledge, no reports have documented CRAO with PAMM following mRNA COVID-19 vaccination. The relationship between COVID-19 vaccination and retinal vascular occlusion remains unknown, highlighting the need for further research.

## Introduction

Ocular complications related to COVID-19 vaccination, such as retinal vascular occlusion and vaccine-induced uveitis, have been documented. Although retinal vascular occlusions have been extensively reported [1], definitive consensus in cohort studies is lacking [2,3], and a clear causal relationship has not been established. Herein, we report a case of central retinal artery occlusion (CRAO) accompanied by paracentral acute middle maculopathy (PAMM) that developed the day after receiving the Pfizer COVID-19 vaccine, with a favorable visual outcome.

## Case presentation

A 33-year-old female presented with a sudden decrease in vision in her left eye one day after receiving the Pfizer COVID-19 vaccine. This was her second or subsequent vaccination. The patient's medical history included schizophrenia and the use of multiple medications, including antipsychotics. She had a smoking history of over ten years and had recently transitioned from traditional cigarettes to heated tobacco. No history of cardiovascular disease, diabetes, pregnancy, or contraceptive use was reported. Blood tests conducted revealed no abnormal findings. Subsequent cardiology evaluations, including echocardiography, did not reveal any signs of thromboembolic risk.

**Figure 1:**
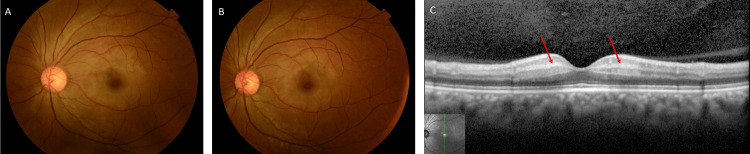
Fundus photography and optical coherence tomography (OCT) at the first visit. (A) Cherry-red spot before treatment. (B) Retinal color tone was improved after treatment. (C) OCT showed a hyper-reflective band spanning the inner nuclear layer (red arrows) and slight retinal thickening.

Her left eye exhibited a diminished direct light reflex, and a cherry-red spot was observed, but the narrowing of the retinal arteries was not clear (Figure 1A). Optical coherence tomography (OCT) revealed high-intensity lesions in the inner nuclear layer (INL) and slight retinal thickening compared with the right eye (Figure 1C). Given the condition's course and the ocular findings, a diagnosis of CRAO accompanied by PAMM was made. Treatment included intravenous D-mannitol, acetazolamide, and a 10-minute ocular massage, resulting in some improvement in subjective symptoms, although her left eye's visual acuity remained unchanged. The retinal appearance seemed to improve (Figure 1B).

**Figure 2:**
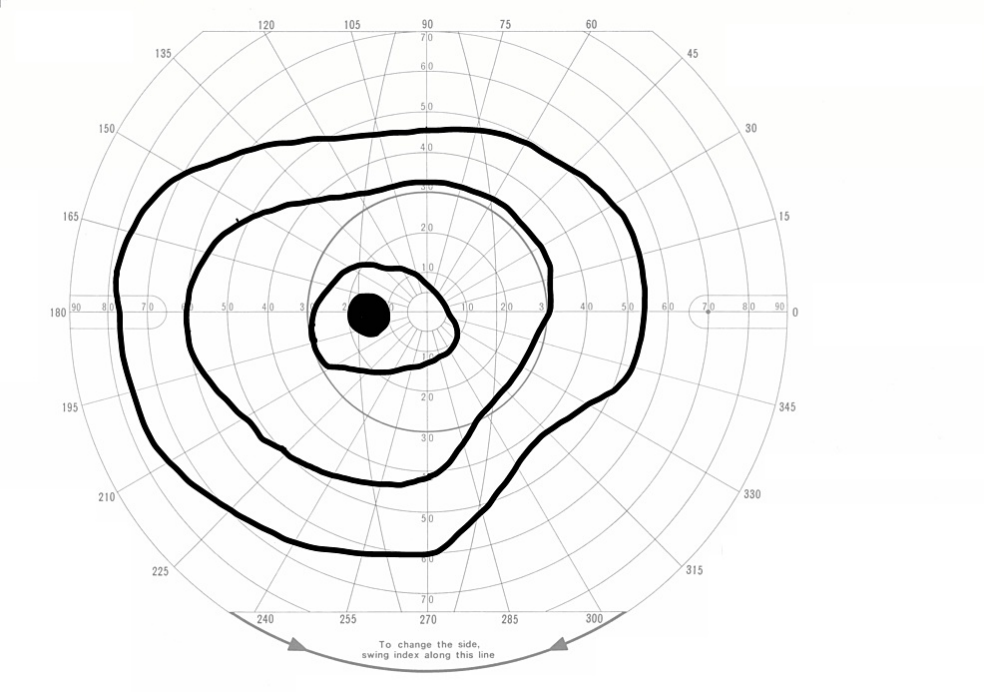
Goldmann visual field test showed slight visual defects in the left eye two days after the first visit.

**Figure 3:**
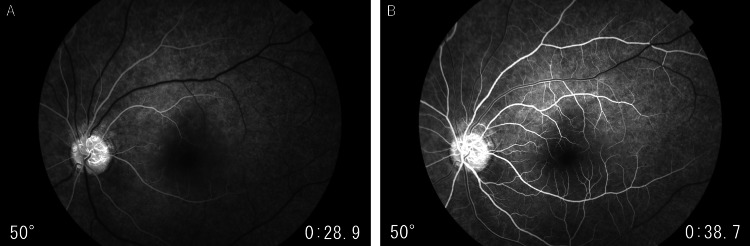
Fluorescein angiography revealed a delay in filling time in the left eye two days after the first visit. (A) Retinal artery filling during the early arteriovenous phase took 28 seconds. (B) Retinal vein filling during the late arteriovenous phase took 38 seconds.

**Figure 4:**
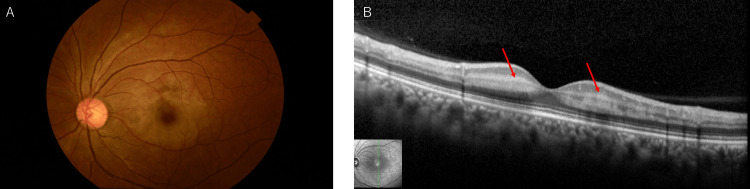
Fundus photography and optical coherence tomography (OCT) two days after the first visit. (A) Retinal color tone showed improvement compared to the initial examination. (B) A hyper-reflective band spanning the inner nuclear layer (INL) showed improvement.

Two days after symptom onset, her corrected visual acuity improved to 0.4. The Goldmann visual field test revealed residual sensitivity loss (Figure 2). Fluorescein fundus angiography did not show retinal vascular occlusion but did indicate a delay in the filling of both the retinal artery and vein (Figure 3). The color tone of the left retina continued to improve (Figure 4A), and OCT indicated the resolution of high-intensity lesions and a reduction in retinal thickness (Figure 4B).

**Figure 5:**
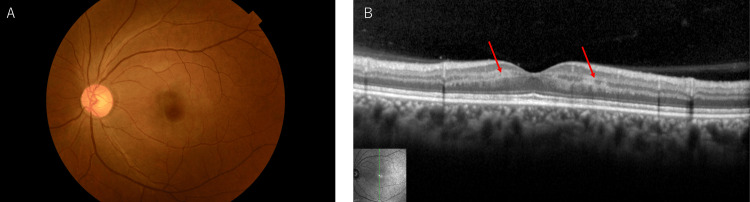
Fundus photography and optical coherence tomography (OCT) 16 days after the first visit. (A) Retinal color tone was almost normalized. (B) A hyper-reflective band spanning the inner nuclear layer (INL) mostly improved, and the retinal thickness was thinning.

Sixteen days after onset, her corrected visual acuity improved to 1.2. The retinal color tone was nearly normalized (Figure 5A), and OCT demonstrated further resolution of the high-intensity lesions and thinning of the retina (Figure 5B).

## Discussion

In this case, a 33-year-old female developed CRAO accompanied by PAMM one day after receiving the Pfizer COVID-19 vaccine, and her clinical course was favorable. Potential risk factors for thrombosis include a history of smoking, the use of antipsychotic medications, and prior COVID-19 vaccination. The patient had recently switched from traditional tobacco to heated tobacco. However, the risk of thrombosis associated with the use of heated tobacco remains uncertain [4]. While antipsychotic medications have been linked to side effects such as pulmonary thromboembolism, no reports have associated them with retinal vascular occlusions [5]. A thorough cardiovascular evaluation did not indicate any thromboembolic risk. The history of smoking constitutes a risk factor for CRAO. However, the onset occurring the day after vaccination raises the possibility of the vaccine being a potential cause of CRAO.

PAMM is characterized by high-intensity lesions in the INL on OCT, likely due to occlusion of the retinal microvasculature. It can be associated with CRAO and typically occurs in the posterior pole, sparing the fovea and often leading to preserved visual acuity [6]. Although the patient's OCT did not exhibit the characteristic high-intensity lesions in the inner retinal layer observed in CRAO, high-intensity lesions were observed in the INL with mild retinal thickening. The initial diagnosis was CRAO, as the patient's visual acuity was limited to hand motions, and a cherry-red spot was observed in the fundus. However, it remains uncertain whether the subsequent improvement in visual acuity was solely attributable to the administered treatment.

Thrombotic occlusion is a significant complication of both COVID-19 and COVID-19 vaccinations. In severe COVID-19 cases, patients may experience elevated inflammatory responses and multi-organ failure [7], leading to a prothrombotic state and thrombotic events [8]. In ophthalmology, several cohort studies suggest a potential relationship between COVID-19 and retinal vascular occlusions [9,10].

Different COVID-19 vaccines require distinct considerations regarding complications. For instance, viral vector vaccines such as AstraZeneca have been associated with vaccine-induced immune thrombotic thrombocytopenia (VITT) and thrombosis with thrombocytopenia syndrome (TTS) [11]. In contrast, mRNA vaccines, such as those from Pfizer, do not contain adenoviral DNA; therefore, they do not cause VITT or TTS [12]. The exact mechanism of vascular occlusion following mRNA vaccination remains unclear; however, it is worth noting that all vaccines may increase the risk of thrombotic events, with viral vector vaccines having a higher reported risk [13]. The association between COVID-19 vaccination and retinal vascular occlusions lacks a definitive conclusion and has mainly been reported in case studies [2,3,14].

Although several reports exist regarding CRAO following mRNA vaccine administration [15,16], none have included details regarding the visual prognosis [14]. A single case report described the occurrence of acute macular neuroretinopathy (AMN) and PAMM following the AstraZeneca COVID-19 vaccination [17]. To the best of our knowledge, this is the first report of CRAO with PAMM following an mRNA COVID-19 vaccination. Additionally, this case represents the first report of a favorable visual outcome in a patient with CRAO following COVID-19 vaccination.

## Conclusions

We report a case of CRAO associated with PAMM occurring one day after receiving Pfizer COVID-19 vaccine, with a favorable clinical outcome. Although an association between COVID-19 vaccines and retinal vascular occlusion has been suggested, the specific mechanisms of retinal vascular occlusion after mRNA vaccination remain unclear. This case provides a valuable example of CRAO with PAMM following mRNA vaccination, with a favorable prognosis. Further research is required to better understand the relationship between COVID-19 vaccines and retinal vascular occlusion.

## References

[1] Sadeghi E, Mahmoudzadeh R, Garg SJ, Nowroozzadeh MH (2023). Ocular posterior segment complications following COVID-19 vaccination. Int Ophthalmol.

[2] Feltgen N, Ach T, Ziemssen F (2022). Retinal vascular occlusion after COVID-19 vaccination: more coincidence than causal relationship? Data from a retrospective multicentre study. J Clin Med.

[3] Napal B, García-Palacios JD, González-Mesones B, Napal JJ, Hernández JL (2023). Retinal vein occlusion in the general population after COVID-19 vaccination and infection. Med Clin (Barc).

[4] (2023). Comprehensive report on research and evidence on novel and emerging tobacco products, in particular heated tobacco products, in response to paragraphs 2(a)-(d) of decision FCTC/COP8(22). https://untobaccocontrol.org/downloads/cop9/main-documents/FCTC_COP9_9_EN.pdf.

[5] Liu Y, Xu J, Fang K (2021). Current antipsychotic agent use and risk of venous thromboembolism and pulmonary embolism: a systematic review and meta-analysis of observational studies. Ther Adv Psychopharmacol.

[6] Moura-Coelho N, Gaspar T, Ferreira JT, Dutra-Medeiros M, Cunha JP (2020). Paracentral acute middle maculopathy-review of the literature. Graefes Arch Clin Exp Ophthalmol.

[7] Siddiqi HK, Mehra MR (2020). COVID-19 illness in native and immunosuppressed states: a clinical-therapeutic staging proposal. J Heart Lung Transplant.

[8] Mir T, Almas T, Kaur J (2021). Coronavirus disease 2019 (COVID-19): multisystem review of pathophysiology. Ann Med Surg (Lond).

[9] Al-Moujahed A, Boucher N, Fernando R, Saroj N, Vail D, Rosenblatt TR, Moshfeghi DM (2022). Incidence of retinal artery and vein occlusions during the COVID-19 pandemic. Ophthalmic Surg Lasers Imaging Retina.

[10] Modjtahedi BS, Do D, Luong TQ, Shaw J (2022). Changes in the incidence of retinal vascular occlusions after COVID-19 diagnosis. JAMA Ophthalmol.

[11] Schultz NH, Sørvoll IH, Michelsen AE (2021). Thrombosis and thrombocytopenia after ChAdOx1 nCoV-19 vaccination. N Engl J Med.

[12] Patel R, Kaki M, Potluri VS, Kahar P, Khanna D (2022). A comprehensive review of SARS-CoV-2 vaccines: Pfizer, Moderna & Johnson & Johnson. Hum Vaccin Immunother.

[13] Hippisley-Cox J, Patone M, Mei XW (2021). Risk of thrombocytopenia and thromboembolism after COVID-19 vaccination and SARS-CoV-2 positive testing: self-controlled case series study. BMJ.

[14] Yeo S, Kim H, Lee J, Yi J, Chung YR (2023). Retinal vascular occlusions in COVID-19 infection and vaccination: a literature review. Graefes Arch Clin Exp Ophthalmol.

[15] Ikegami Y, Numaga J, Okano N, Fukuda S, Yamamoto H, Terada Y (2022). Combined central retinal artery and vein occlusion shortly after mRNA-SARS-CoV-2 vaccination. QJM.

[16] Girbardt C, Busch C, Al-Sheikh M (2021). Retinal vascular events after mRNA and adenoviral-vectored COVID-19 vaccines - a case series. Vaccines (Basel).

[17] Vinzamuri S, Pradeep TG, Kotian R (2021). Bilateral paracentral acute middle maculopathy and acute macular neuroretinopathy following COVID-19 vaccination. Indian J Ophthalmol.

